# Adjunctive Dental Procedures and Pain Assessment Among a Group of Patients Attending Dental University Hospital at King Saud University: A Cross-Sectional Study

**DOI:** 10.7759/cureus.44809

**Published:** 2023-09-06

**Authors:** AlBandary H AlJameel, Abdulaziz M Alshehri, Saud H Alzuhair, Mohammed H Al masud, Abdulaziz J Alasmri, Yazeed A Alkhunefer, Nassr S Almaflehi

**Affiliations:** 1 Periodontics and Community Dentistry, College of Dentistry, King Saud University, Riyadh, SAU; 2 Restorative Dental Science Department, King Saud University, College of Dentistry, Riyadh, SAU; 3 Dentistry, College of Dentistry, King Saud University, Riyadh, SAU; 4 Periodontics and Community Dentistry, King Saud University, Riyadh, SAU

**Keywords:** pain assessment, numerical rating scale, adjunctive dental procedures, discomfort, pain

## Abstract

Background/purpose

A large percentage of people still experience discomfort and pain during dental visits, even with advancements in instrumental techniques that enable dentists to manage patients in a pain-free manner. The aim of this study was to assess the prevalence and levels of pain induced by various dental procedures, including adjunctive dental procedures.

Material and methods

A structured, custom-made questionnaire composed of 20 questions written in Arabic with accompanying pictures of instruments to simplify instrument identification for the patients was designed. The questionnaire was designed to investigate and determine the factors that cause pain and discomfort during dental procedures and was introduced to adult patients attending their dental appointments at the College of Dentistry. All data were collected using an online link that was distributed to patients attending dental clinics at the Dental University Hospital at King Saud University either through their mobile phones or the researcher's tablet. All data were entered and analyzed using SPSS version 25.

Results

A total of 158 patients participated in the study. The findings revealed that 50% of participants experienced pain from mouth mirror retraction and 28.48% experienced pain from suction. Participants also reported that the most common instruments associated with pain were the ultrasonic scaler (88.57%) and the periodontal probe (87.88%).

Conclusion

The findings provided valuable insights into the prevalence of pain during dental procedures and the factors that may contribute to this experience. Adjunctive dental procedures appeared to be causing a high prevalence of pain that could be avoided if dentists/dental assistants were more aware of it. These findings may have important implications for dental practitioners looking to reduce pain and improve patient experience during the provision of dental care.

## Introduction

One of the most prevalent concerns that people present within the hospital is pain [1]. The definition of pain differs according to the medical field [2]. In general, pain is defined by the International Association for the Study of Pain (IASP) as a "sensorial and emotionally unpleasant experience that promotes behavioural changes in a person, often impeding normal daily activities" [3]. Furthermore, Mersky described pain as "an unpleasant sensory and emotional experience with actual or potential tissue damage or described in terms of such damage" [4].

From a dental perspective, orofacial pain originating from the dental or adjacent structures is referred to as dental pain. Multiple diseases, such as dental caries, periodontal disease, trauma, occlusal dysfunction, and abscesses, can cause such pain [5]. A large percentage of people still experience discomfort and pain during dental procedures, even with advancements in instrumental techniques that have enabled dentists to manage patients in a pain-free manner [6].

Pain could be a barrier to oral-health-related quality of life when it is not properly controlled [7]. Moreover, pain elimination is not simple to achieve due to various uncontrolled factors that may contribute to the pain experience, such as past traumatic dental clinic experiences, dental fear, and anxiety [8].

Most previous studies investigated the occurrence and management of dental pain before and/or after the provision of dental procedures, in addition to some studies that aimed to assess the impact of anxiety and/or fear on dental pain [1,2,5,9-13]. In contrast, studies of different factors that contribute to the pain experienced during dental procedures are not well reported in the literature.

In clinical practice, determining pain severity and locating its source are among the standard techniques. As a result, pain assessment instruments are used to assess pain intensity as well as track the effectiveness of and reaction to treatment provided (just a suggestion to re-order some words if you think it's needed: as well as track the reaction and effectiveness of treatment provided) [14].

One of the most prevalent concerns that people present within the hospital is pain [1]. The definition of pain differs according to the medical field [2]. In general, pain is defined by the IASP as a "sensorial and emotionally unpleasant experience that promotes behavioural changes in a person, often impeding normal daily activities" [3]. Furthermore, Mersky described pain as "an unpleasant sensory and emotional experience with actual or potential tissue damage or described in terms of such damage" [4].

From a dental perspective, orofacial pain originating from the dental or adjacent structures is referred to as dental pain. Multiple diseases, such as dental caries, periodontal disease, trauma, occlusal dysfunction, and abscesses, can cause such pain [5]. A large percentage of people still experience discomfort and pain during dental procedures, even with advancements in instrumental techniques that have enabled dentists to manage patients in a pain-free manner [6].

Pain could be a barrier to oral-health-related quality of life when it is not properly controlled [7]. Moreover, pain elimination is not simple to achieve due to various uncontrolled factors that may contribute to the pain experience, such as past traumatic dental clinic experiences, dental fear, and anxiety [8].

Most previous studies investigated the occurrence and management of dental pain before and/or after the provision of dental procedures, in addition to some studies that aimed to assess the impact of anxiety and/or fear on dental pain [1,2,5,9-13]. In contrast, studies of different factors that contribute to the pain experienced during dental procedures are not well reported in the literature.

In clinical practice, determining pain severity and locating its source are among the standard techniques. As a result, pain assessment instruments are used to assess pain intensity as well as track the effectiveness of and reaction to treatment provided. Just a suggestion to re-order some words if you think it is needed, as well as track the reaction and effectiveness of the treatment provided. [14].

Guivarc'h et al. investigated dental students’ attitudes towards the management of pain and anxiety during a dental emergency and found that the vast majority of students always inquired about the patient's pain before treatment, while intraoperative pain was given less attention [15]. It was also reported that drilling, anaesthetic injection, and extraction are the three most feared dental procedures [16]. Studies have also indicated that root canal treatment and tooth extraction are associated with significant pain and discomfort [17].

The aim of this study was to investigate the different factors that can cause pain or discomfort during the provision of various dental treatments. This investigation is not limited to the main dental procedures performed by dentists; it also accounts for some adjunctive dental procedures, such as the use of suction or mouth mirrors. By assessing and classifying these factors, a valuable clinical recommendation can be provided to reduce pain and/or discomfort experienced by patients in dental clinics and consequently enhance the provision of dental care.

The importance of assessing the frequency and severity of pain caused by various dental procedures, including adjunctive dental procedures, and testing the premise that adjunctive dental procedures can cause pain while providing dental care can help dentists understand the type of pain caused by adjunctive procedures, which can affect the patient experience in the dental clinic.

The objective of this study was to look into the numerous things that could lead to discomfort or pain while doing various dental procedures. This inquiry takes into account several auxiliary dental operations, such as the use of suction or mouth mirrors, in addition to the primary dental procedures carried out by dentists. By evaluating and categorizing these variables, an insightful clinical proposal can be made to lessen patients' pain and/or suffering at dental offices and, as a result, improve the delivery of dental care.

Specific aims: (i) to determine the prevalence of pain induced by various dental procedures, including adjunctive dental procedures; (ii) to determine the level of pain induced by various dental procedures; and (iii) to test the hypothesis that adjunctive dental procedures can cause pain during the provision of dental care.

## Materials and methods

Ethical considerations

This study was approved by the Institutional Review Board (IRB) of King Saud University with registration no. E-22-6841. Consent was obtained from each patient before their participation.

Study sample

A total of 158 participants were interviewed at the Dental University Hospital at King Saud University in Riyadh, the capital city of Saudi Arabia. The participants were subjected to a questionnaire designed to assess some factors that might induce pain during the provision of dental treatment/care, including adjunctive dental procedures.

A structured, custom-made questionnaire composed of 20 questions written in Arabic was designed to assess the pain sources and levels experienced by dental patients; some pictures were used to simplify the questions for the participants. The authors of this study were responsible for delivering a Google Form containing the questionnaire immediately after each dental procedure to control the patient's recall bias. The sample was selected based on the exclusion and inclusion criteria.

Inclusion criteria were as follows: (i) regular attendance at the dental clinic; (ii) ≥18 years old; (iii) no concomitant medical conditions as determined via medical history; (iv) no unsatisfactory experiences during his or her past visits to dental clinics; and (v) the ability to understand, read, and complete the questionnaire.

Exclusion criteria were as follows: (i) unwillingness to participate in this study; and (ii) needing to undergo an emergency dental procedure.

Questionnaire

Several tools for pain assessment have been developed [18]. Pain assessment instruments are used to assess pain prevalence and intensity, as well as track the effectiveness of and reaction to treatment decisions [14]. The Numerical Rating Scale (NRS), developed by Downie et al. in 1978 [19], was used in this questionnaire, which is denoted by vertical or horizontal lines with a total of 11 values ranging from 0 to 10, with 0 representing no pain, 1 to 3 representing mild pain, 4 to 6 representing moderate pain, and 7 to 10 representing severe pain [19].

The questionnaire was composed of 20 questions, starting with consent to participation. Then, three questions on demographics (nationality, age, and sex) were asked, followed by a question asking if you had visited the dental clinic before or not and, if so, how many times. The next two questions were similar to each other: the first one asked about the reason for this dental visit, while the other was about the specialty of the procedure performed during the visit. The remaining 13 questions were all about pain induced by the 13 included instruments, in this order: suction, mouth mirror, periodontal probe, ultra-sonic scaler, curette, handpiece, clamp, Tofflemire matrix/wedge, impression, retraction cord, dental files, elevators, and forceps.

Statistical analysis

Descriptive statistics (mean, median, standard deviation, and percentage) are displayed in the graphs and tables. For inferential statistics, with normality and equality of variance assumptions satisfied, a one-way analysis of variance (ANOVA) test was used, followed by Tukey as a post hoc test. However, a standard advanced statistical analysis was used if needed, and the level of significance was set at a P-value of <0.05. The collected data were inserted into IBM SPSS Statistics version 25.0 for statistical analysis.

## Results

The study included 158 participants who were treated at the Dental University Hospital at King Saud University in Riyadh, with ages ranging from 18 to 70 years. The majority of participants were male (127), while 31 of them were female. The majority of participants were Saudi (81%), while only 19% were non-Saudi. Participants were asked to complete a questionnaire regarding their experience of pain during various dental procedures.

**Figure 1:**
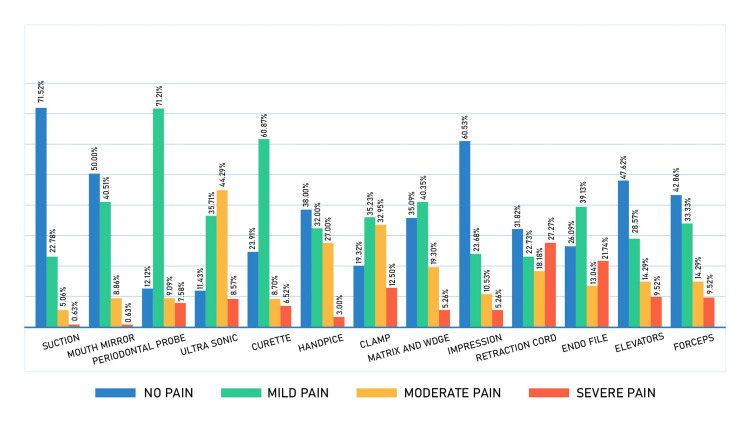
Pain distribution of all 13 selected instruments in the study

Figure 1 shows the distribution of pain and its severity among the selected dental instruments and procedures. As shown in the figures, the most reported sources of severe pain were the retraction cord (27.3%), followed by the endofile (21.74%), and the clamp (12.50%). Suction was the least painful source (71.5% reported no discomfort), followed by impression (60.53%) and a mouth mirror (50%). A very high percentage of participants reported that the periodontal probe (71.2%) and curette (60.9%) caused them moderate levels of pain. The most common instruments associated with pain at all levels were the ultrasonic scaler and periodontal probe, with 88.57% and 87.88% of participants reporting pain, respectively.

**Figure 2:**
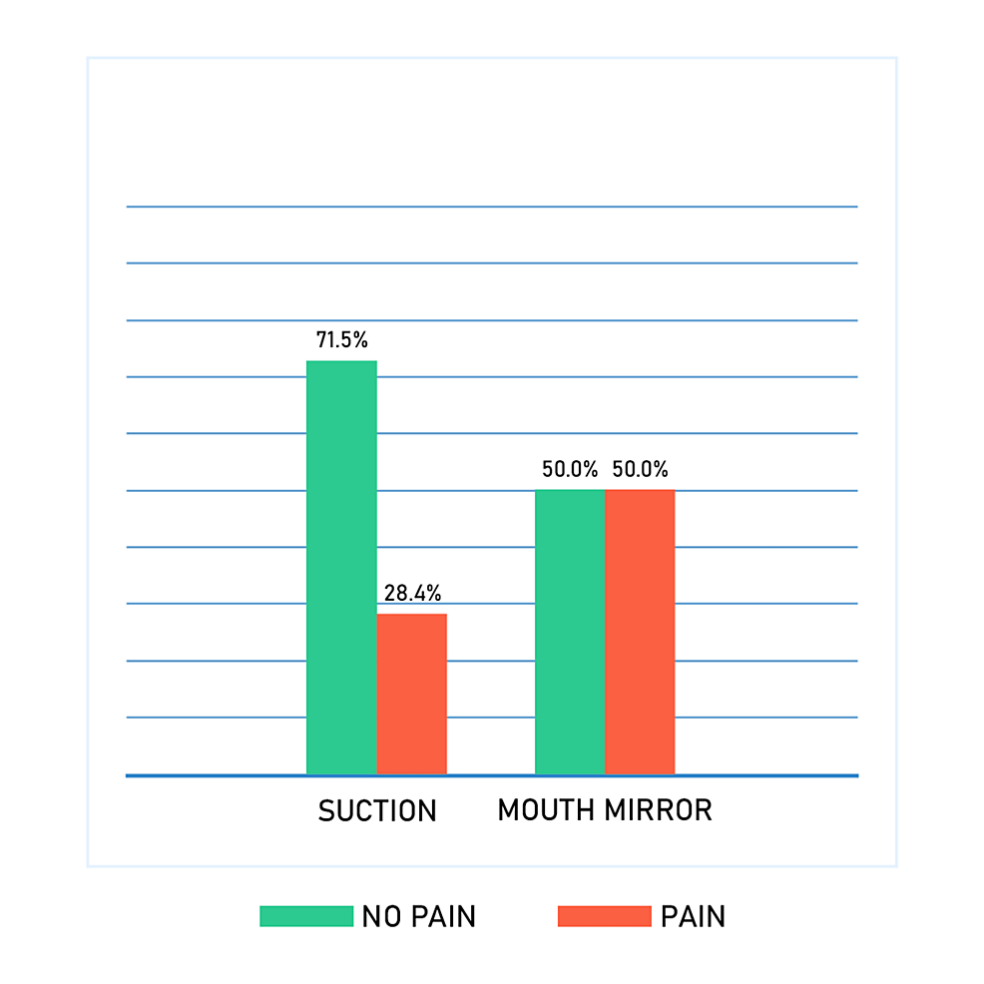
Pain prevalence of mirror and suction

When participants were asked about sources of pain from adjunctive dental procedures, interestingly, the findings revealed that 50% of participants experienced pain from mouth mirror retraction and 28.48% experienced pain from suction, as shown in Figure 2.

The association between different demographic variables and sources of pain was explored, and the findings revealed significant associations between some patient demographics and specific dental procedures. For example, it was found that nationality was significantly associated with clamp placement (P=0.0036) and dental files (P=0.008), and Saudis reported higher levels of pain (Table 1).

**Table 1:** Pain distribution by nationality among all 13 selected instruments in the study

Factor	Instrument	Nationality	Level								Chi-square	P-value
			No pain		Mild		Moderate		Severe			
			n	%	n	%	n	%	n	%		
Nationality	Suction	Saudi	91	71.1%	29	22.7%	7	5.5%	1	0.8%	0.475	0.924
		Non-Saudi	22	73.3%	7	23.3%	1	3.3%	0	0.0%		
	Mouth mirror	Saudi	64	50.0%	53	41.4%	10	7.8%	1	0.8%	1.205	0.752
		Non-Saudi	15	50.0%	11	36.7%	4	13.3%	0	0.0%		
	Periodontal probe	Saudi	7	14.0%	36	72.0%	4	8.0%	3	6.0%	1.565	0.667
		Non-Saudi	1	6.3%	11	68.8%	2	12.5%	2	12.5%		
	Ultra-sonic scaler	Saudi	7	12.7%	18	32.7%	25	45.5%	5	9.1%	1.180	0.758
		Non-Saudi	1	6.7%	7	46.7%	6	40.0%	1	6.7%		
	Curette	Saudi	7	21.2%	20	60.6%	3	9.1%	3	9.1%	1.560	0.668
		Non-Saudi	4	30.8%	8	61.5%	1	7.7%	0	0.0%		
	Handpiece	Saudi	30	34.9%	28	32.6%	25	29.1%	3	3.5%	3.093	0.378
		Non-Saudi	8	57.1%	4	28.6%	2	14.3%	0	0.0%		
	Clamp	Saudi	12	16.0%	25	33.3%	29	38.7%	9	12.0%	8.539	0.036
		Non-Saudi	5	38.5%	6	46.2%	0	0.0%	2	15.4%		
	Tofflemire matrix/wedge	Saudi	16	32.0%	21	42.0%	11	22.0%	2	4.0%	4.155	0.245
		Non-Saudi	4	57.1%	2	28.6%	0	0.0%	1	14.3%		
	Impression	Saudi	16	53.3%	8	26.7%	4	13.3%	2	6.7%	3.353	0.340
		Non-Saudi	7	87.5%	1	12.5%	0	0.0%	0	0.0%		
	Retraction cord	Saudi	6	33.3%	4	22.2%	3	16.7%	5	27.8%	0.217	0.975
		Non-Saudi	1	25.0%	1	25.0%	1	25.0%	1	25.0%		
	Dental files	Saudi	1	6.3%	7	43.8%	3	18.8%	5	31.3%	11.717	0.008
		Non-Saudi	5	71.4%	2	28.6%	0	0.0%	0	0.0%		
	Elevators	Saudi	7	50.0%	4	28.6%	2	14.3%	1	7.1%	0.300	0.960
		Non-Saudi	3	42.9%	2	28.6%	1	14.3%	1	14.3%		
	Forceps	Saudi	8	57.1%	3	21.4%	2	14.3%	1	7.1%	4.036	0.258
		Non-Saudi	1	14.3%	4	57.1%	1	14.3%	1	14.3%		

Results also showed that sex was significantly associated with clamp placement (P=0.05), and males reported a higher prevalence of pain than females, as shown in Table 2.

**Table 2:** Pain distribution by sex among all 13 selected instruments in the study

Factor	Instrument	Sex	Level								Chi-square	P-value
			No pain		Mild		Moderate		Severe			
			n	%	n	%	n	%	n	%		
Sex	Suction	Male	92	72.4%	28	22.0%	6	4.7%	1	0.8%	0.622	0.891
		Female	21	67.7%	8	25.8%	2	6.5%	0	0.0%		
	Mouth mirror	Male	62	48.8%	53	41.7%	11	8.7%	1	0.8%	0.694	0.875
		Female	17	54.8%	11	35.5%	3	9.7%	0	0.0%		
	Periodontal probe	Male	6	10.5%	41	71.9%	6	10.5%	4	7.0%	2.027	0.567
		Female	2	22.2%	6	66.7%	0	0.0%	1	11.1%		
	Ultra-sonic scaler	Male	6	9.8%	23	37.7%	27	44.3%	5	8.2%	1.657	0.647
		Female	2	22.2%	2	22.2%	4	44.4%	1	11.1%		
	Curette	Male	10	26.3%	22	57.9%	4	10.5%	2	5.3%	2.218	0.528
		Female	1	12.5%	6	75.0%	0	0.0%	1	12.5%		
	Handpiece	Male	29	37.2%	25	32.1%	22	28.2%	2	2.6%	0.478	0.924
		Female	9	40.9%	7	31.8%	5	22.7%	1	4.5%		
	Clamp	Male	10	14.3%	28	40.0%	22	31.4%	10	14.3%	7.814	0.050
		Female	7	38.9%	3	16.7%	7	38.9%	1	5.6%		
	Tofflemire matrix/wedge	Male	15	32.6%	20	43.5%	8	17.4%	3	6.5%	2.162	0.540
		Female	5	45.5%	3	27.3%	3	27.3%	0	0.0%		
	Impression	Male	18	60.0%	7	23.3%	3	10.0%	2	6.7%	0.585	0.900
		Female	5	62.5%	2	25.0%	1	12.5%	0	0.0%		
	Retraction cord	Male	7	36.8%	4	21.1%	3	15.8%	5	26.3%	1.763	0.623
		Female	0	0.0%	1	33.3%	1	33.3%	1	33.3%		
	Dental files	Male	4	23.5%	5	29.4%	3	17.6%	5	29.4%	4.560	0.207
		Female	2	33.3%	4	66.7%	0	0.0%	0	0.0%		
	Elevators	Male	6	42.9%	5	35.7%	1	7.1%	2	14.3%	3.450	0.327
		Female	4	57.1%	1	14.3%	2	28.6%	0	0.0%		
	Forceps	Male	4	30.8%	6	46.2%	1	7.7%	2	15.4%	5.115	0.164
		Female	5	62.5%	1	12.5%	2	25.0%	0	0.0%		

Furthermore, it was found that the number of dental visits, especially "periodontal treatment-related visits", was significantly associated with pain as a result of curette use (P=0.003), while a weak association was found for ultrasonic scaler use (P = 0.06) (Table 3).

**Table 3:** Pain distribution by number of visits among all 13 selected instruments in the study

Factor	Instrument	Number of visits	Level								Chi-square	P-value
			No pain		Mild		Moderate		Severe			
			n	%	n	%	n	%	n	%		
Number of visits	Suction	No visit	2	100.0%	0	0.0%	0	0.0%	0	0.0%	3.023	0.963
		1–3 visits	13	61.9%	7	33.3%	1	4.8%	0	0.0%		
		4–6 visits	23	69.7%	8	24.2%	2	6.1%	0	0.0%		
		>6 visits	75	73.5%	21	20.6%	5	4.9%	1	1.0%		
	Mouth mirror	No visit	2	100.0%	0	0.0%	0	0.0%	0	0.0%	11.260	0.258
		1–3 visits	13	61.9%	4	19.0%	4	19.0%	0	0.0%		
		4–6 visits	14	42.4%	18	54.5%	1	3.0%	0	0.0%		
		>6 visits	50	49.0%	42	41.2%	9	8.8%	1	1.0%		
	Periodontal probe	No visit	0	100.0%	0	0.0%	0	0.0%	0	0.0%	6.661	0.353
		1–3 visits	2	18.2%	7	63.6%	0	0.0%	2	18.2%		
		4–6 visits	0	0.0%	11	91.7%	1	8.3%	0	0.0%		
		>6 visits	6	14.0%	29	67.4%	5	11.6%	3	7.0%		
	Ultra-sonic scaler	No visit	0	0.0%	0	0.0%	0	0.0%	1	100.0%	16.365	0.060
		1–3 visits	3	25.0%	5	41.7%	3	25.0%	1	8.3%		
		4–6 visits	0	0.0%	4	30.8%	8	61.5%	1	7.7%		
		>6 visits	5	11.4%	16	36.4%	20	45.5%	3	6.8%		
	Curette	No visit	0	0.0%	0	0.0%	0	0.0%	1	100.0%	25.268	0.003
		1–3 visits	4	40.0%	5	50.0%	0	0.0%	1	10.0%		
		4–6 visits	4	57.1%	3	42.9%	0	0.0%	0	0.0%		
		>6 visits	3	10.7%	20	71.4%	4	14.3%	1	3.6%		
	Handpiece	No visit	2	100.0%	0	0.0%	0	0.0%	0	0.0%	8.404	0.494
		1–3 visits	5	45.5%	3	27.3%	3	27.3%	0	0.0%		
		4-6 visits	9	40.9%	7	31.8%	4	18.2%	2	9.1%		
		>6 visits	22	33.8%	22	33.8%	20	30.8%	1	1.5%		
	Clamp	No visit	2	100.0%	0	0.0%	0	0.0%	0	0.0%	14.413	0.108
		1–3 visits	0	0.0%	3	33.3%	4	44.4%	2	22.2%		
		4–6 visits	3	15.8%	10	52.6%	5	26.3%	1	5.3%		
		>6 visits	12	20.7%	18	31.0%	20	34.5%	8	13.8%		
	Tofflemire matrix/wedge	No visit	1	100.0%	0	0.0%	0	0.0%	0	0.0%	4.985	0.836
		1–3 visits	1	20.0%	3	60.0%	1	20.0%	0	0.0%		
		4–6 visits	3	27.3%	6	54.5%	2	18.2%	0	0.0%		
		>6 visits	15	37.5%	14	35.0%	8	20.0%	3	7.5%		
	Impression	No visit	0	100.0%	0	0.0%	0	0.0%	0	0.0%	4.127	0.659
		1–3 visits	3	100.0%	0	0.0%	0	0.0%	0	0.0%		
		4–6 visits	2	50.0%	2	50.0%	0	0.0%	0	0.0%		
		>6 visits	18	58.1%	7	22.6%	4	12.9%	2	6.5%		
	Retraction cord	No visit	0	100.0%	0	0.0%	0	0.0%	0	0.0%	7.423	0.283
		1–3 visits	0	0.0%	0	0.0%	1	50.0%	1	50.0%		
		4–6 visits	0	0.0%	2	66.7%	0	0.0%	1	33.3%		
		>6 visits	7	41.2%	3	17.6%	3	17.6%	4	23.5%		
	Dental files	No visit	0	100.0%	0	0.0%	0	0.0%	0	0.0%	6.946	0.326
		1–3 visits	2	66.7%	1	33.3%	0	0.0%	0	0.0%		
		4–6 visits	2	28.6%	4	57.1%	0	0.0%	1	14.3%		
		>6 visits	2	15.4%	4	30.8%	3	23.1%	4	30.8%		
	Elevators	No visit	0	100.0%	0	0.0%	0	0.0%	0	0.0%	5.040^a^	0.539
		1–3 visits	1	33.3%	1	33.3%	1	33.3%	0	0.0%		
		4–6 visits	3	100.0%	0	0.0%	0	0.0%	0	0.0%		
		>6 visits	6	40.0%	5	33.3%	2	13.3%	2	13.3%		
	Forceps	No visit	1	100.0%	0	0.0%	0	0.0%	0	0.0%	4.079	0.906
		1–3 visits	1	33.3%	1	33.3%	1	33.3%	0	0.0%		
		4–6 visits	2	66.7%	1	33.3%	0	0.0%	0	0.0%		
		>6 visits	5	35.7%	5	35.7%	2	14.3%	2	14.3%		

## Discussion

The study aimed to assess the prevalence of pain provoked by various dental instruments and procedures, including adjunctive dental procedures, among adults attending regular dental visits at the Dental University Hospital at King Saud University.

Using the collected data, the order of pain prevalence among all the instruments included was as follows: ultrasonic scaler (88.57%), periodontal probe (87.88%), clamp (80.68%), curette (76.09%), endo files (73.91%), retraction cord (68.18%), Tofflemire matrix/wedge (64.91%), handpiece (62%), forceps (57.14%), elevators (52.38%), mouth mirror (50%), impression (39.47%), and suction (28.4%). Notably, three of the instruments with the highest prevalence of pain were related to the provision of periodontal treatment/care, which is often used in nonsurgical procedures without local anaesthesia. It is important for students and dentists to use these instruments carefully, as they can induce pain and discomfort. By improving their technique and being mindful of their patients' comfort, they can enhance their overall dental experience.

The results revealed that endodontic instruments were found to cause a noticeable level of pain, which is expected, as these types of procedures often occur when the patient is experiencing pain prior to the visit and is more likely to report higher levels of pain. Next, the retraction cord is mentioned, but it is unclear why it would be one of the most pain-inducing procedures since this type of procedure should not be painful with the administration of local anaesthesia. The results also showed that restorative instruments such as handpieces and Tofflemire matrices/wedges should not cause pain since they are used after administering local anaesthesia. Following surgical instruments, there may be a misconception that the pressure from such instruments is painful, but this cannot be entirely avoided.

In this study, pain induced by adjunctive dental procedures (such as suction and retraction with a mouth mirror) along with impressions were also explored, which are not supposed to be painful since the main function of each one is not to penetrate any tissues. Applying excessive, unnecessary pressure when using adjunctive dental procedures could be an explanation for pain while concentrating on the main procedures, such as preparation or extraction. There were not enough studies in the literature exploring this area with which to compare these findings. We must raise awareness regarding this issue among all kinds of practitioners, from students to consultants, to enhance the experience of patients in dental clinics.

In the most severe pain category, the two highest instruments among the 13 instruments assessed were retraction cords (27.27%) and endo files (21.74%). As mentioned earlier, students and dentists must be aware of the importance of administering local anaesthesia with the application of retraction cords. This study also found that severe pain caused by endofiles was consistent with previous studies [20,21], as Bhardwaj et al. stated that all shaping procedures caused postoperative pain that decreased with time [20]. Likewise, Kashefinejad et al. stated that patients treated under hand files (endo files) had high postoperative pain [21]. It should be noted that the abovementioned studies tried to explore pain as a result of endofiles after the provision of dental treatment, while this study explored the patient’s experience of pain during the provision of such treatment.

Regarding the ultra-sonic scaler being the most painful among all levels of pain in this study, this is consistent with a previous study by Braun et al., who conducted a study to compare the ultra-sonic system to another system and showed that the ultra-sonic scaler had a high pain score [22].

There were not enough studies found in the literature that discussed this topic and explored pain caused by instruments and adjunctive dental procedures during the provision of dental treatment/care in particular; therefore, it was difficult to compare all the findings. It is highly recommended that further studies be conducted to investigate this topic. It is also crucial to teach dental students during clinical courses the importance of performing adjunctive dental procedures gently to enhance patients’ experiences.

Limitations

Our study had fewer females than males; therefore, it would be better to conduct future studies with a more balanced sample. There were not enough studies in the literature discussing the study topic, which consequently did not allow a sufficient comparison of the study findings. It is also important to note that the assessment of pain is subjective, and there might be other factors that contribute to the occurrence of pain and its intensity.

The results of this study showed that adjunctive dental procedures might cause avoidable and undesirable pain that can impact the patients' experience negatively. Therefore, dentists and dental assistants can help make the dental experience more comfortable for patients and raise clinic standards for patient care, thus improving the quality of dental care and improving the oral health of the population by providing a painless experience, which can affect the frequency of dental visits and improve oral health.

## Conclusions

Overall, the findings provided valuable insights into the prevalence of pain during dental visits and some factors that may contribute to this experience. Adjunctive dental procedures such as using mouth mirrors and suction were found to be associated with a high prevalence of pain; therefore, it is important for dental professionals to assess the cause of the pain and try to manage it in order to improve patients' experiences. It is also important to emphasize to dental students how important it is to execute adjunctive dental procedures with care. These findings may have important implications for dental practitioners seeking to reduce pain and improve the overall patient experience during a dental visit.
